# A case of secondary pulmonary alveolar proteinosis, but prior to myelodysplastic syndrome

**DOI:** 10.1002/rcr2.29

**Published:** 2013-10-31

**Authors:** Yunfeng Zhao, Wei Xiong, Xueling Wu

**Affiliations:** 1 Department of Respiratory Disease, Shanghai Pudong New Area Gongli HospitalShanghai, China; 2 Institute of Respiratory Medicine, Xinqiao Hospital, Third Military Medical UniversityChongqing, China

**Keywords:** Myelodysplastic syndrome, secondary pulmonary alveolar proteinosis

## Abstract

Pulmonary alveolar proteinosis (PAP) is a rare lung disorder. We herein report the first case of PAP that happened 2 years before myelodysplatic syndrome (MDS). A 34-year-old Chinese presented with a developed recurrent cough and shortness of breath. Computed tomography scan disclosed ground-glass opacities with interlobular septal thickening. Histological examination showed eosinophilic dense homogenous material filling in the alveolar. This precipitate had a fine granular appearance. The eosinophilic material was periodic acid–Schiff reaction-positive. The patient was diagnosed with PAP. Two years later he was admitted to a hospital because of dizziness of 1-month duration. Hematological examination showed white blood cells was 2700, hemoglobin was 7.4 g/dL, and platelet count was 21,000 platelets/mm^3^. Following bone marrow biopsy and histopathologic examination, he was diagnosed with MDS with refractory anemia and excess blasts. So for PAP patients, follow-up tests should be considered in order to find any possible underlying disease.

## Introduction

Pulmonary alveolar proteinosis (PAP) is a rare lung disorder characterized by excessive accumulation of surfactant-derived lipoproteins within the pulmonary alveoli, causing mild dyspnea to severe respiratory distress. PAP accompanied by other diseases is generally regarded as secondary PAP, of which the most frequent underlying disease is hematological malignancy. The longest interval from myelodysplastic syndrome (MDS) to PAP is 12 years; herein we report the first case of PAP that happened 2 years before MDS.

## Case Report

**Figure 1:**
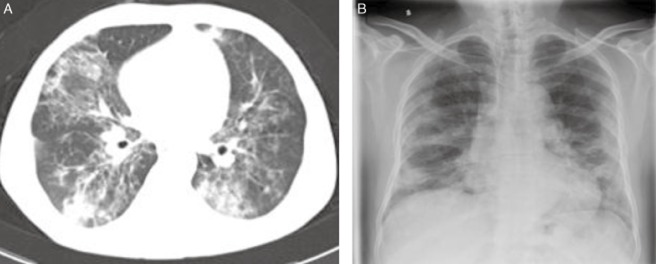
(A) Posteroanterior chest radiograph showing symmetric, perihilar ground-glass, and reticulonodular opacities. (B) Chest computed tomography scan containing scattered patches of ground-glass opacities with thickened interlobular septums.

**Figure 2:**
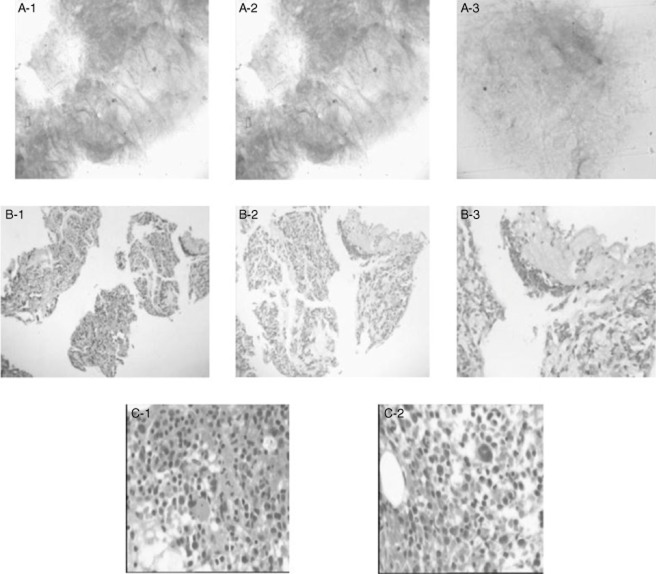
(A) The bronchoalveolar lavage fluid filled with periodic acid–Schiff (PAS)-positive materials (A-1: PAS stain × 100; A-2: PAS stain × 200; A-3: PAS stain × 400). (B) Pathological findings of transbronchial lung biopsy demonstrated that eosinophilic dense homogenous materials filled the alveolar and the alveolar septa are rather thickened by lymphocyte infiltration in the right middle lobe (B-1: hematoxylin and eosin [HE] stain × 100; B-2: HE stain × 200; B-3: HE stain × 400). (C) Pathological findings of bone marrow biopsy showed that the myeloid to erythroid precursors (M : E) ratio was 1 : 1, there was more red pulp in part of bone marrow pulp which leads to the ratio of 1.5 : 1. Three-line hematopoietic cells and erythrocytes in different stages of maturation existed. Promyelocytes scattered solely. There were 2–5 meg per bone pits and no hyperplasia of the fiber existed.

A 34-year-old male Chinese who had no significant past medical history or any congenital disease manifested a developing recurrent cough and gradually deteriorating shortness of breath in February 2008. His body temperature was normal and bilateral fine crackles were audible at the back of the patient. Routine blood test showed the white blood cells (WBCs), hemoglobin and platelet were all normal. Chest X-ray showed infiltrative opacity in bilateral lungs (Fig. 1A). Arterial blood gas analysis under indoor air revealed hypoxemia and hypocapnia. The patient was then started on antibiotics because there was no improvement in the computed tomography (CT) scan, which disclosed ground-glass opacities with interlobular septal thickening and signs of volume loss in bilateral lungs (Fig. 1B). Lung function test revealed diffuse capacity of the lungs for carbon monoxide was reduced moderately. Bronchoalveolar lavage fluid showed a light-milky appearance and was periodic acid–Schiff (PAS) reaction-positive (Fig. 2A). Histological examination showed eosinophilic dense homogenous material precipitation, which demonstrated a fine granular appearance filling in the alveolar. The eosinophilic material was PAS reaction-positive (Fig. 2B). Based on these evidences, the patient was diagnosed with PAP and was discharged because of his refusal for further treatment.

The patient was admitted to a hospital again in February 2010 because of dizziness of 1-month duration. Anemia was noticed in the palpebral conjunctiva without any rash eruption being observed on the skin or mucosa. Bilateral fine crackles were still audible at the back, also the spleen was palpable. Routine blood test showed WBCs was 2700, hemoglobin was 7.4 g/dL and platelet was 21,000/mm^3^. Abdominal ultrasound and CT scan showed hypersplenotrophy. The patient was eventually diagnosed with MDS with refractory anemia and excess blasts by means of bone marrow biopsy and histopathologic examination. (Fig. 2C).

## Discussion

PAP was first reported by Rosen et al. in 1958. Three categories of PAP have been described: congenital, secondary, and acquired. Secondary PAP results from other underlying causes, which include hematologic malignancies, pharmacologic immunosuppression, certain infections, and the inhalation of inorganic dust (silica) or toxic fumes; however, etiology remains controversial. F. Bonella et al. discovered that PAP induced by dust inhalation was also autoimmune and that there were cases when some drugs could induce PAP [1]. Among all these cases, hematologic malignancies were the most frequent underlying diseases [2]. The incidence of secondary PAP has been estimated to be 5.3% among all patients with hematologic malignancies.

Secondary PAP earlier than MDS is uncommonly encountered with unclear mechanism. It has been reported that the content of pulmonary alveoli in patients with PAP consists of a complex of phospholipids and proteins, which are usually synthesized by type II alveolar cells and cleared by pulmonary macrophages. Insufficient clearance of phospholipids by pulmonary macrophages may promote the development of PAP. Dirksen et al. suggested that alveolar macrophages might be derived from the malignant clone itself, and in some circumstances have been shown to carry specific defects that may explain the observed functional impairment of surfactant clearance [3]. This suggestion is supported by the alleviation of the pulmonary process following the recovery of hematopoietic function. Further studies are required to resolve the genesis of secondary PAP with hematologic malignancies.

Secondary PAP usually occurs after MDS; only one case was reported showing that PAP happened at the same time as MDS. In this article, we report the first case in the world of PAP associated with, but preceding, MDS that was diagnosed by way of histological findings of transbronchial lung biopsy. In the literature, the reported median duration of symptoms prior to diagnosis is 7 months, male-to-female ratio is 2.65 : 1, and percentage of smokers is 72% [4]. In our case, the patient was 34 years old when he was diagnosed with PAP; however, there was no potential underlying disease existing then. The CT image of “crazy-paving,” defined as a network of smoothly thickened reticular (septal) lines superimposed on areas of ground-glass opacity (GGO), is the common hallmark of PAP. Areas of crazy-paving in PAP are typically widespread and bilateral. In one study, in a secondary PAP group, GGOs typically showed a diffuse pattern, whereas it showed a patchy geographic pattern in the autoimmune PAP group [5]. The diffusion of GGO in PAP patients, which indicates secondary PAP, possibly calls for a high degree of suspicion for potential primary diseases.

A previous report found that significant self-confinement of PAP occurred in 7.9% of patients, and the median time from diagnosis to resolution was 20 months [6]. However, the treatment for secondary PAP mainly depends on the therapy used for the underlying disease. Whole lung lavage has been used to ameliorate symptoms; however, this method was not used in this case because of the patient's refusal owing to the mild symptoms when he was diagnosed with PAP. Allogeneic bone marrow transplantation could also be a successful therapy for PAP secondary to MDS.

In summary, we present an unusual case of secondary PAP associated with, but preceding, MDS that has not been reported previously as far as we know. In conclusion, for PAP patients, follow-up tests should be considered in order to find any possible underlying primary disease.

## References

[1] Bonella F, Bauer PC, Griese M (2011). Pulmonary alveolar proteinosis: new insights from a single-center cohort of 70 patients. Respir. Med.

[2] Trapnell BC, Whitsett JA, Nakata K (2003). Pulmonary alveolar proteinosis. N. Engl. J. Med.

[3] Dirksen U, Hattenhorst U, Schneider P (1998). Defective expression of granulocyte-macrophage colony-stimulating factor/interleukin-3/interleukin-5 receptor common beta chain in children with acute myeloid leukemia associated with respiratory failure. Blood.

[4] Seymour JF, Presneill JJ (2002). Pulmonary alveolar proteinosis: progress in the first 44 years. Am. J. Respir. Crit. Care Med.

[5] Ishii H, Trapnell BC, Tazawa R (2009). Comparative study of high-resolution CT findings between autoimmune and secondary pulmonary alveolar proteinosis. Chest.

[6] Seymour JF, Presneill JJ (2002). Pulmonary alveolar proteinosis: progress in the first 44 years. Am. J. Respir. Crit. Care Med.

